# Social Entrepreneur’s Psychological Capital, Political Skills, Social Networks and New Venture Performance

**DOI:** 10.3389/fpsyg.2020.00925

**Published:** 2020-06-10

**Authors:** Li Xin Guo, Chi-Fang Liu, Yu-Sheng Yain

**Affiliations:** ^1^School of Business, Huaiyin Institute of Technology, Huai’an, China; ^2^Department of Business Administration, Cheng Shiu University, Kaohsiung, Taiwan

**Keywords:** social entrepreneur, political skill, psychological capital, social network, new venture performance

## Abstract

Scholars have begun to realize the importance of entrepreneurial political skills to new ventures. Namely, social entrepreneurship is a context, in which entrepreneurs expend great efforts in networking politically to integrate diverse resources and share interests (e.g., ecology wellness) for sustainability. In this paper, we integrate the social exchange theory and the resource-based view to discuss how social entrepreneurs’ political skills enhance new ventures’ performance through their social network (size/diversity and structural holes), and discuss how psychological capital in entrepreneurial contexts can influence new venture performance by political skills’ functionality. By connecting significant entrepreneurship research constructs at different levels, this article not only enriches our knowledge about the ways in which social entrepreneurs’ political skills and psychological capital affect the performance of ventures, but also offers new ventures some guidance on how to use political skill to improve their social networking and performance. Implications for social entrepreneurial sustainability are discussed.

## Introduction

New ventures contribute to the socioeconomic development. Therefore, understanding the key influencing factors of new ventures’ performance has very important theoretical and practical value (Baumol and Strom, 2007). Entrepreneurship researchers have repeatedly emphasized the importance of entrepreneurial social networks to the survival and development of new ventures (Shane and Cable, 2002; Hoang and Antoncic, 2003; Ruef et al., 2003; Li and Zhang, 2007; Chen et al., 2017), having found that entrepreneurs often cultivate and build extensive social networks to get the various resources they need (Eisenhardt and Schoonhoven, 1996; Stuart and Sorenson, 2007; Stam and Elfring, 2008; De Carolis et al., 2009).

Although existing literatures have found significant positive effects of social networks on venture performance, one fundamental question remains unanswered: why do some entrepreneurs construct and develop social networks to enhance new ventures’ performance better than others? From the behavioral perspective, studies have argued that an entrepreneur’s skills can impact the construction and usage of social networks. Although many studies have examined the direct effects of social networks on venture performance, few studies have analyzed the indirect effects of social networks. For example, social networks act as a mediation variable that might transform entrepreneurial skill’s effect into venture performance.

In the context of social entrepreneurship, efforts to answer such questions are still wanting. Social entrepreneurship is a special type of entrepreneurship, not only for pursuing economic return but also for pursuing the goal of solving social/public problems with alignment between diverse interests. In such contexts, political skills are important for entrepreneurs. Ferris et al. (2012) claim that individuals with higher political skills are more likely to build good relationships with others and to occupy central positions in the workplace (Treadway et al., 2013). Omrane (2015) argues that individuals can use appropriate skills to build good social networks that will help them access important social resources. Fang et al. (2015) have proposed that entrepreneurs with high political skills can build more stable and more adaptive social networks than those with low ones.

All of these pioneering studies have commonly pointed out a shared research direction – we need to know more about how entrepreneurs’ political skill influences their social network and in turn, venture performance. Moreover, little is known regarding whether, and how, the four major dimensions of political skill differentially affect the functionality of social networks to improve new venture performance. Although scholarly works have extensively examined the influences of psychological capital on organizational results, it is suggested that the studies focusing on the psychological capital’s consequent influences on entrepreneurial performance, through a series of mediating factors, are very limited. Studies examining the relationship between psychological capital, political skill and social networks in the entrepreneurship literature are also very limited. Considering that political skill is related to power relations in the organization, which demands a strong psychological base, it comes to mind that this set of skills may be related to psychological capital.

Further, the success of social entrepreneurship is inseparable from extensive social support and help. Because pursuing both social and economic goals often leads to a shortage of resources for social ventures, social entrepreneurs need to propagate their social mission and stimulate the prosocial behavior of external resource owners. When social entrepreneurs persuade resource owners to provide support and help, they must be able to positively influence the psychological state of others in order to gain others’ empathy and social identity. Thus, entrepreneurial psychological capital may play an important role in this process, and we believe that entrepreneurs with a positive mental state may be better able to leverage their political skills and social networks. In the process of interpersonal interaction, individual emotional states usually affect each other, thus entrepreneurs’ self-confidence, optimism, hope and resilience may affect others’ emotional state, help them earn the trust of others, and finally, may increase the probability of success in social entrepreneurship.

**FIGURE 1:**
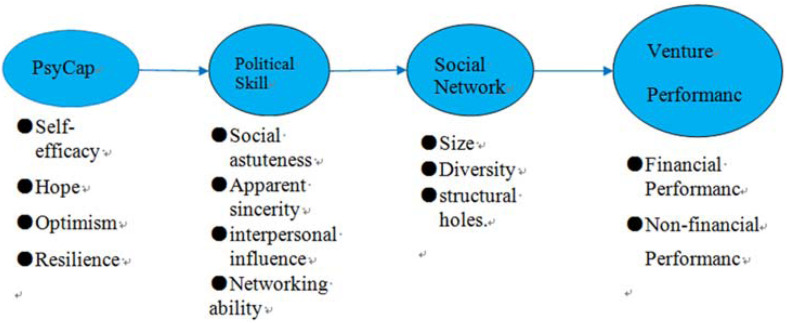
Conceptual scheme.

In sum, to discuss the above questions, we conceptually elaborate the potential influences of psychological capital on political skills. Following this, we go into an in-depth discussion of the effects of political skills on an entrepreneur’s social network size, diversity, structural holes, and on their new venture’s financial performance. We discuss the different effects of the four dimensions of political skills. Furthermore, the mediation roles of the above-mentioned social network dimensions are explored. Moreover, the special context of social entrepreneurship is suitable for a good illustration of the issues proposed here. Our study will contribute to network-based entrepreneurship research literatures and firm-level political skill research literatures, especially in the context of social entrepreneurship. Before we start the discussions of the interrelationships, we sketch the conceptual scheme in Figure 1.

## Theoretical Background and Proposition Development

### Psychological Capital and Political Skills

The concept of positive organizational behavior, which can be described as the reflection of positive psychology in organizational life, contributed to the conceptualization of psychological capital (Avey et al., 2008, 2009; Luthans et al., 2008). Psychological capital could be perceived as a holistic and positive mental state that a collective of individuals share in an organization.

There are various definitions of psychological capital, related to the positive psychological state of an individual’s and/or a collective’s development. In one of these definitions, psychological capital is considered as the state of having the confidence, or in other words, self-efficacy to provide the necessary effort to succeed in difficult tasks. According to another definition, psychological capital is a positive expectation about the possibility of success in the present and future, or in other words, the state of being optimistic. In addition, psychological capital is defined as the ability to be determined to reach, and to move toward new ways to achieve success, or in other words, to carry hope. Finally, it can be said that psychological capital is conceptualized as one’s ability to recover when faced with problems or possible troubles and to show an insistent attitude during such times, i.e., to be persistent or to be durable Luthans et al. (2006a,b).

Based on these different, but highly interrelated conceptualizations, scholars have agreed that psychological capital has four basic dimensions: self-efficacy, optimism, hope and resilience Luthans et al. (2007). Self-efficacy is a faithful belief a person owns, to control the events and solve the problems one faces in organizational life, with the positive motivation and cognitive resources benefiting future prospects Stajkovic and Luthans (1998). It is also defined as the individual’s positive belief toward the abilities he has while doing and working Stajkovic and Luthans (1998). Optimism can be defined as good expectations for the future (Carver and Scheier, 2002a,b). At the core of such definition, individuals relate positive events as a result of internal, permanent and common causes Seligmanand Csikszentmihalyi, 2000. Hope, the third dimension of psychological capital, interacts with goal-oriented energy that arises from a plan to meet specific goals with a sense of accomplishment Snyder (2000). Finally, resilience is seen as the person’s ability to cope with many negative situations (such as obstacles or uncertainty) to re-bounce or to eventually lead to success (Luthans et al., 2006b). It is a state in which positive change and development are displayed in the face of adverse situations such as difficulty, uncertainty, conflict, failure, etc. Masten (2001), Luthans (2002) act. Erkus and Findikli (2013).

The relationship between psychological capital and organizational outcomes has been widely investigated from various angles in the literature. It has been stated that it provides organizations with a competitive advantage. It has further been suggested that psychological health might be associated with political skills both positively and negatively (Bedi and Skowronski, 2014). However, the detailed cause-effect relationships have been under-discussed. That said, although knowledge about how an individual’s political skills might affect another person’s psychological state has been accumulated, few have explored the ways in which a person’s positive psychological state could influence his/her own political skills’ functionality, especially in an entrepreneurial context Luthans et al. (2004).

During a new venture, an entrepreneur’s, as well as other employees’ psychological capital may have a positive impact on their performance. However, their way of influence may be different. For example, the psychological capital of an entrepreneur may directly and positively affect the functionality of his/her political skills, enabling him/her to better motivate internal employees and build better social networks, thereby positively affecting new venture performance. Nevertheless, other employees’ psychological capital may make them identify with the new venture and be willing to contribute more to it, which may have a positive impact on new venture performance. Intuitively, people’s emotions can interact and influence each other, thus, the psychological capital between entrepreneurs and other employees may actually affect the interactions people have with one another, and other employees’ psychological capital may indirectly affect the functionality of the entrepreneur’s political skills. To avoid confusion, this study only discusses the impact of entrepreneurial psychological capital on the functionality of political skills and new venture performance.

Reasonably based on the discussion above, the political skills used to achieve people’s organizational goals are likely to be influenced by psychological capital, which refers to the qualities of individuals to reveal their strengths. Therefore, in this study, the relationship between psychological capital and political skill is proposed.

***P0: Psychological capital in entrepreneurial contexts could influence new venture performance through functional factors (e.g., the political skills and social networks in this article).***

***P1: Psychological capital of an entrepreneur or an entrepreneurial team could positively influence the functionality of political skills.***

### Political Skill and Social Network

Existing research suggests that people’s personality and ability characteristics affect the construction of their social networks (Douglas and Ammeter, 2004; Wei et al., 2012; Ferris et al., 2017; Burt et al., 2018). Though many political skill scholars in organization behavior have repeatedly mentioned the important role of individual political skill in the process of interpersonal action in the organizational context (Ferris et al., 2005, 2007), we still do not conclusively know why different political skill levels could lead to different network structure/attributes.

The existence of strong and mutually beneficial exchange ties can promote the mobilization of social network resources (Fombrun, 1982; Nahapiet and Ghoshal, 1998). The social exchange theory suggests that human relations are based on rational choice (Thibaut and Kelley, 1959; Homans, 1961) and constitute a resource-exchange relationship – an exchange of goods, both material and non-material, such as the symbols of approval or prestige. The theory also assumes that actors achieve their goals by exchanging resources with others, and these goals are often beyond their reach (Lawler and Thye, 1999). Here, resource exchange can be regarded as social exchange. The main aspect of social exchange is that it takes place between individuals who have the characteristics of self-interest and mutual need – two or more actors have what the other needs, and they can independently decide whether, and how much, to exchange (Aldrich and Zimmer, 1986). Persons who give much to others try to get equally as much from them (reciprocity), and persons who get much from others are under pressure to give equally as much back. This process of influence tends to work out at equilibrium in the exchanges (Homans, 1961). Appraisal of whether or not a new relationship is attractive and valuable takes place through a comparison of the ratio of the rewards and costs (which is calculated by their self-standards) of the connection (Thibaut and Kelley, 1959). Following one person’s initial contact with another person, the formation and maintenance of the relationship depends on the rewards/costs ratio/level they have experienced or expected. Individuals’ behavior in the social interaction process is often highly selective – they seek to produce the most satisfactory results, and once they discover that they cannot reach their goals, they will choose to give up the relationship (Thibaut and Kelley, 1959). The core idea of social exchange theory is that two actors voluntarily exchange their own resources. However, there are also involuntary exchange relationships, for example, ones lacking a good trading partner, taking place when the actors are not satisfied with the partner but have to maintain the existing exchange relationship. Drawing on the above social exchange theory, logic dictates that the traits and definitions of political skill determine that self-interested actors, with a variety of private goals, will usually prefer to be in contact or exchange resources with politically skilled individuals for three reasons: the first is that politically skilled individuals are more likely to discover and identify others’ needs and interests due to their social astuteness and savviness, so politically skilled individuals can make an impression to others suggesting that they own some resources needed by others, by sending appropriate signals in social situations. This may effectively attract others’ attention and interest. The second reason is that politically skilled individuals are more likely to gain others’ trust as they are, or appear to be, sincere, genuine and honest. These traits signal to others that being in contact, or exchanging resources with such individuals is safe and beneficial. The third reason is that politically skilled individuals are more likely to make favorable impressions to others, suggesting that they can provide the needed resources or help to others due to their interpersonal influence ability. To interpret the above logic in detail, we discuss the ways in which the four dimensions of political skill influence social networks size, diversity, and structural holes, as follows:

#### Social Astuteness and Social Network

Social astuteness means that people with high political skill can better understand the actions and motivations of others in social interactions (Ferris et al., 2007). This competence helps politically skilled individuals to discover others’ potential interest points and needs in social settings, and to know how to behave or speak/talk to others, which can attract others’ attention and let them believe that building a personal relation with the individual is beneficial to their needs. It is difficult for low social astuteness individuals to attract other people’s attention and interest or to construct a connection as they cannot understand others’ behavioral motivation and significance well and do not have sufficient knowledge of others’ hidden interests and needs. Based on these arguments, we propose that:

***P2a: Social astuteness has a positive relation with the size of entrepreneurial networks for both strong and weak ties.***

High social astuteness individuals, as astute observers of others, can more effectively search for and discover valuable information in a variety of social settings, know their existing ties, which can provide the necessary types of resources, and know the types of resources which are lacking, as well as the types which are redundant. This results in a greater likelihood of establishing a connection with the persons than low social astuteness individuals, as previously argued. Thus, we assume that:

***P2b: Social astuteness has a positive relation with the diversity of entrepreneurial networks for both strong ties and weak ties.***

In comparison to the other three dimensions of political skill, social astuteness can endow entrepreneurs the ability to discover and identify valuable information/resources from other individuals or organizations and help them know who they can build ties with and how. Although apparent sincerity, interpersonal influence, and networking ability can also be helpful for building ties, the ties or resource-exchanging cannot occur if individuals cannot discover or identify which relationships are worth building. Social astuteness helps entrepreneurs to find more potential relationship objects, and as such, we expect that:

***P2c: The relationship between the social astuteness and entrepreneurial networks’ size/diversity will be stronger than the relationships between the other three dimensions of political skill and entrepreneurial networks’ size/diversity.***

#### Apparent Sincerity and Social Network

Apparent sincerity means that people with high political skills can make themselves appear genuine (Ferris et al., 2007). A person who pursues a connection with another person often has the basic confidence that the specific relationship will result in some material or spiritual benefits for him/her (resource exchange and reciprocity), while the other person will not harm his/her interests by leveraging his/her flaws and limitations (non-opportunistic behavior). High apparent sincerity individuals can make a good impression to others, suggesting to them that they are trusted, sincere, genuine, and that they will not leverage others’ limitations for self-interest and bring risk or harm to others. Therefore, most people prefer to establish friendships and collaborative ties with high apparent sincerity individuals and dislike to be in contact with deceitful and selfish people, interaction with whom results in more costs and fewer rewards. At the possibility of a better alternative, people will select to construct ties with more apparent sincerity individuals, thus we argue that:

***P3a: Apparent sincerity has a positive relation with the size and diversity of entrepreneurial networks for both strong ties and weak ties.***

In a weak and inefficient institutional system environment, there is a general lack of trust among people, and this environment is conducive to social entrepreneurs to build good social networks. Such social networks, with many structural holes, can complement the shortcomings of formal institutions to a certain extent (Batjargal et al., 2013), and can help social entrepreneurs to obtain resources and support that are not available within the formal institutional framework (Burt, 1992; Batjargal, 2007). For example, entrepreneurial networks with structural holes help ventures get new information, knowledge, loans, venture capital, and emotional support (Granovetter, 1973; Burt, 1992). In this institutional context, entrepreneurs have a stronger motivation to establish networks rich in structural holes. Entrepreneurs who are, or appear to be, honest and forthright can more easily win others’ trust, act as a broker, or bridge ties between persons who lack a sufficient trust or lack a direct connection than low apparent sincerity entrepreneurs. This reasoning suggests that:

***P3b: Apparent sincerity has a positive relation with entrepreneurial networks’ structural holes.***

#### Interpersonal Influence and Social Network

Interpersonal influence refers to the idea that politically skilled individuals have an unassuming and convincing personal style that exerts a powerful influence on others around them and allows people to adapt and calibrate their behavior to different situations to elicit the desired responses from others (Ferris et al., 2007). Individuals with high interpersonal influence can adjust their behaviors according to different situations to influence others to produce favorable behaviors (Ferris et al., 2005). Because of the new venture’s liability of newness (Fombrun, 1982), lack of necessary resources and legitimacy, new entrepreneurs have a strong motivation to build network ties. Social astuteness may help entrepreneurs find who possesses the necessary resources for new ventures, while apparent sincerity may help them acquire the owned-resource persons’ trust and leave a good impression. However, whether the owned-resource persons agree to build and maintain an exchanging-resources relationship with entrepreneurs depends on others’ perception of the type of resources the entrepreneur has, as well as their willingness to exchange. Generally, strong ties are more conducive to the exchange and utilization of network resources (Fombrun, 1982; Nahapiet and Ghoshal, 1998). Individuals with high interpersonal influence are more likely to influence and control the choices of others (Ferris et al., 2005, 2007). The main principle of social exchange theory is that interdependence and mutual obligations determine the form of their relationship (Cropanzano et al., 2014). High interpersonal influence entrepreneurs know how to delegate the appropriate help or resources to the resource-owned persons according to their different personalities and needs, leading to the desired response (the owned-resource persons give the needed or necessary help/resources toward the new venture). As such, high interpersonal influence entrepreneurs can more easily establish and maintain exchanging-resources relationships with the owned-resource persons or organizations than low interpersonal influence entrepreneurs. Based on these arguments, we assume that:

***P4: Interpersonal influence has a positive relation with the size and diversity of entrepreneurial networks for both strong ties and weak ties.***

#### Networking Ability and Social Network

Networking ability means that individuals with high political skills are able to identify and establish various connections, such as friendships or alliances with others (Ferris et al., 2007). In a transition context, where the formal institutional system is weak, a resource-rich and interdependent network can bring sustained competitive advantage to a new venture. Ventures with many alliances are more powerful than lone-ventures, or ones with little alliance, in super-competitive environments. As social exchange theory outlines, resource-exchanging and interdependence are at the basis of a relationship. High networking ability entrepreneurs can effectively search, scan, discover and identify the resource-owned persons or organizations, find the common interest points and resource-exchanging possibilities, and then develop diverse contacts with these individuals or organizations in further building strong, beneficial alliances. Based on this discussion, we propose that:

***P5a: Networking ability has a positive relation with the size and diversity of entrepreneurial networks for both strong ties and weak ties.***

As argued above, entrepreneurs who have a network rich in structural holes can act as a broker and bridge ties. The brokers generate returns each time they broker an exchange (Renzulli et al., 2000). In this way, entrepreneurs can act as intermediaries between suppliers and customers and benefit from them (Burt, 1992). Further, bridging ties can promote entrepreneurs in having greater access to resources (Stam and Elfring, 2008; Batjargal, 2010). Having a wide range of resource channels can help entrepreneurs get resources at any given time (Batjargal et al., 2013), thus, entrepreneurs have a strong motivation to construct social networks rich in structural holes, because highly networked entrepreneurs are more likely to build friendships and interest alliances (Ferris et al., 2007). High networking ability entrepreneurs can also search and discover more disconnected network clusters and can establish bigger and more diverse networks rich in structural holes than low networking ability entrepreneurs. Thus, we propose that:

***P5b: Networking ability has a positive relation with entrepreneurial networks’ structural holes.***

### Social Network and New Venture Performance

Many researchers agree that social entrepreneurship is embedded in social networks (Aldrich and Zimmer, 1986) (pp. 3–23). The literature clearly indicates that entrepreneurs often obtain resources through their personal social relationships (Adler and Kwon, 2002). Entrepreneurs’ social networks can help them identify business opportunities (Batjargal, 2010), activate resources (Batjargal and Liu, 2004), and gain legitimacy (Elfring and Hulsink, 2003). From the resource-based view, entrepreneurs’ personal networks can be conceived as new ventures’ special resources that can bring competitive advantage to ventures. Network size or sum of connections with other persons and organizations with resources needed for ventures can influence or determine whether new ventures can acquire sufficient resource from external networks with the ventures development. A larger network may provide more valuable and rare resources than a smaller one, and may better meet the new ventures’ resource demand. Therefore, a different network size may lead to a different venture performance. Due to this reasoning, we suggest that:

***P6a: Entrepreneurial networks’ size will be positively related to both new ventures’ financial and non-financial performance.***

Entrepreneurs’ social networks usually include different types and characteristics of network members, and different types of members often have different types of resources. Therefore, the diversity of entrepreneurs’ networks will affect the scope of their access to resources (He et al., 2013). New ventures usually have higher uncertainty and entrepreneurs often cannot know the types and channels of resources needed in the future. Therefore, they need networks with higher diversity (Elfring and Hulsink, 2007). Because networks with higher diversity can reduce this uncertainty, new ventures can obtain various resources and help through different types of network members (Renzulli et al., 2000). In addition, a higher social network diversity can reduce the cost of searching for resources (McEvily and Zaheer, 1999). If the diversity of the entrepreneur’s social network is low, the entrepreneur needs to spend more time searching for resources from other networks (He et al., 2013). Thus, the more diverse the entrepreneur’s social network, the more it can expand their horizons and increase the reputation of the venture among the network members (Podolny, 2001). The whole impact of network diversity on a new venture will have a positive effect on its performance. Based on the above analysis, we make the following proposition:

***P6b: Entrepreneurial networks’ diversity will be positively related to both new ventures’ financial and non-financial performance.***

In a social network with few structural holes, information and resources are usually shared by most members (Granovetter, 1995). In contrast, in a social network with more structural holes, information and resources are often not fully shared, and entrepreneurs have an advantage in obtaining those informal resources and information (Burt, 1992; Batjargal, 2007). For example, entrepreneurs can get early access to information about new products, new markets and new channels (Batjargal et al., 2013) as they can play the role of long-distance network bridge connections (Stam and Elfring, 2008; Batjargal, 2010; Ferris et al., 2012). In the entrepreneur’s social network, mutual trust among members may also generate customer recommendations (Granovetter, 2000). In social networks with more structural holes, entrepreneurs can also play an intermediary role, such as connecting suppliers and buyers (Burt, 1992), which also gives them access to more diverse resources (Stam and Elfring, 2008; Batjargal, 2010). If entrepreneurs can obtain more raw materials, funds and technology, then they can choose more ambitious strategies, continue to develop new products and expand market space, all of which will increase long-term returns for new ventures (Batjargal and Liu, 2004). The extensive existence of structural holes gives entrepreneurs more choice and freedom. Entrepreneurs can obtain resources through separated network clusters and they do not need to compromise with powerful members in order to obtain resources (Burt, 1992). The existence of structural holes results in isolated network members in the entrepreneur’s social network, which will encourage entrepreneurs to obtain different emotional support (Batjargal et al., 2013). For example, relatives, classmates, friends, and partners of entrepreneurs are usually isolated from each other. They can provide different social and emotional support to satisfy entrepreneurs’ needs (Churchill et al., 1987). These types of emotional support increase entrepreneurs’ confidence and help them focus on improving new venture performance (Krueger and Dickson, 1994). If entrepreneurs can integrate network clusters that are far apart, this can lead to some positive results, such as promoting the integration of different knowledge and resources, establishing alliances, and obtaining venture capital (Granovetter, 1973; Batjargal, 2007). These integrations are likely to bring rapid revenue growth to ventures (Batjargal et al., 2013). Summarizing these arguments, we propose that:

***P6c: Entrepreneurial networks’ structural holes will be positively related to both new ventures’ financial and non-financial performance.***

### Psychological Capital, Political Skill, Social Networks, and New Venture Performance

As previously mentioned by some scholars – business is a political arena (Gray and Ariss, 1985; Mintzberg and Waters, 1985; Jackall, 1988). The conception of political skills is derived from this idea and it is defined as the ability to understand and influence the behavior of others (Ahearn et al., 2004; Ferris et al., 2005, 2007). Because of the new venture’s liability of newness (Stinchcombe and March, 1965), lack of necessary resources and legitimacy, entrepreneurs need to be able to persuade resource holders to provide the support they need (Aldrich and Martinez, 2001; Choi and Shepherd, 2005; Rutherford and Buller, 2007). For example, entrepreneurs need to be able to motivate employees to work hard, attract customers to buy their own services or products, and persuade financiers to provide loans (Tocher et al., 2012). Thus, it is not just the entrepreneur’s social network that is important, but the ability of entrepreneurs to use and manage the network is also important for ventures to acquire resources (Batjargal, 2006; Jack et al., 2008). The entrepreneur’s political skill can be conceived as an important resource for its key role in convincing other individuals or organizations to provide valuable and necessary resources for ventures. For example, Ahearn et al. (2004), Breland et al. (2007), and Semadar et al. (2006) have found that individuals with higher political skills are better able to extract resources from social networks than those with lower ones. Batjargal’s (Barney, 1991) research results show that entrepreneurs with higher network capabilities can get their new ventures better recognized by the public. Fang et al. (2015) case study found that entrepreneurs with higher political skills can better build their own social networks, make better use of network relationships to obtain resources, and improve new venture performance. Barney (1991) proposes that rare, irreplaceable and heterogeneous resources and abilities can bring sustainable competitive advantage. This includes various tangible and intangible assets, such as organizational practices and processes, knowledge and information, management capabilities and company culture (Barney, 1991; Barney et al., 2001). RBV has emphasized the importance of the skills of top managers (Castanias and Helfat, 1991; Maritan, 2001). If entrepreneurs who own high political skills can be viewed as a specific, rare and valuable resource, we expect that politically skilled entrepreneurs will bring better performance for their new ventures than politically unskilled entrepreneurs. Further, we define an entrepreneur as a new ventures’ founder and an actual operator and controller. Entrepreneurs also substantially take on the role of CEO; in fact, there is significant empirical support for CEO effect on firm performance. For example, some studies have found that CEO personality differences can lead to significant differences in company performance. Quigley and Hambrick (2014) found that the CEO effect on company performance(ROS) had increased from 8.6 percent (1950–1969) to 20.3 percent (1970–1989) to 26.4 percent (1990–2009). More recently, Tocher et al. (2012) have found that political skills have a significant and positive influence on new venture performance (Tocher et al., 2012). Existing research finds that political skills primarily affect non-financial performance (Tocher et al., 2012). However, it remains unclear whether political skills also affect financial performance. Because there are many indicators for measuring company performance (Stam et al., 2014), different measurement indicators may bring different results. Therefore, we need to further study the impact of political skills on new venture financial performance. As we all know, in transition economy and social environments, there exist resource-flow barriers due to weak and inefficient institutions. Entrepreneurial resources are often constrained and have low liquidity, and entrepreneurs’ ability to obtain resources from external environments becomes an important source of competitive advantage. Most researchers accept that entrepreneur political skill is an important competence contributing to the acquisition of the necessary resources to enhance new venture performance in a weak institution context. This argument may have both theoretical and practical legitimacy.

Overall, based on the logic of Fang et al. (2015) and Tocher et al. (2012), we believe that the political skills of entrepreneurs will help entrepreneurs to establish good relationships with resource owners and promote mutually beneficial resource exchanges. Thus, politically skilled entrepreneurs will establish and develop better social networks, which are richer in resources, and then use these networks to achieve a better performance. Drawing on the above arguments, we propose that:

***P7: Influenced by psychological capital, entrepreneur political skill is positively related to both new ventures’ financial and non-financial performance, through the functional effects of social networks (size/density).***

## Implications and Future Research Directions

### Implications for Social Entrepreneurship Sustainability

New venture performance is a critical condition for the sustainability of entrepreneurship. This does not only mean that entrepreneurs and their newly founded ventures would survive over time, but it also means that the entrepreneurial efforts could be constantly committed into their business. Our Perspective paper argues that, through active efforts in exercising political skills to be effective, new venture performance could be achieved on and on, by continuously expanding (i.e., the size) and broadening (i.e., the diversity) social networks with resource embeddedness. This paper also implies that merely having political skills may not be fully effective, unless such skills really function on the development of social networks. Therefore, we further incorporated psychological capital into the traditional research framework and proposed that psychological capital may affect the functionality of the entrepreneur’s political skills, social networks, and new venture performance.

On the other hand, this study also has important theoretical and practical implications for social entrepreneurship and social innovation. Unlike the benefits exchange in the business context, in the context of social entrepreneurship, entrepreneurs are more likely to obtain resources through charitable donations or volunteer services from others. For resource providers who have not received the corresponding benefits, whether they are willing to help social ventures depends on whether they accept the social goals of social ventures, or whether they have sufficient confidence in the success of social entrepreneurship. Therefore, in the context of social entrepreneurship, the psychological capital of entrepreneurs may play a more important role in helping social ventures obtain resources. This is due to the fact that the psychological capital of entrepreneurs may not only affect the functionality of their political skills but also the psychological and emotional states of resource providers or members of social networks, which will in turn affect the resource acquisition of social ventures. In today’s world, there are more and more social problems, such as unemployment, poverty, environmental pollution, prevention and treatment of infectious diseases, and aging of the population etc. These social problems have threatened sustainable development and cannot be solved by relying only on traditional government and market mechanisms. Thus, continuous social innovation is required. Like social entrepreneurship, the success of social innovation also requires reaching broad social consensus and building public confidence. Therefore, the psychological capital of social innovators is also very important, because their positive psychological states may positively affect the emotions of various social groups and win their support and help.

### Limitations and Future Research Directions

Limited by research resources and time, this paper only provides a conceptual framework and a series of theoretical propositions, and these theoretical assumptions have not been empirically tested. Future research may empirically examine the proposed and related hypotheses with multiple and combined methodologies. For example, to examine the conceptual framework in this article, studies need to adopt both multivariate statistics, social network analysis, and financial modeling methods. A high demand of the data collection and testing skills is obvious. In addition, because the research concept of this study involves multiple levels of individuals, teams or groups and organizations, in future empirical research, it may be necessary to further select a suitable cross-level theoretical foundation and build a suitable theoretical model. In data analysis, cross-level modeling and data analysis methods may be required, such as HLM, Mplus, etc.

In this paper, we mainly discuss the impact of entrepreneurs’ own psychological capital on the functionality of political skills, as other people’s psychological capital may have different effects on the functionality of entrepreneurial political skill. Future research can further analyze the impact of psychological capital of internal colleagues and external social network members on the functionality of entrepreneurial political skill.

Further, social entrepreneurship generally has a higher risk of failure. When social entrepreneurs encounter entrepreneurial failures, it is vital for them to be able to recover from the spiritual shock they experience. They need to be encouraged and comforted, and the cause of failure needs to be clearly analyzed. Therefore, future research can explore and develop some new research frameworks. For example, we can study the relationships between psychological capital, resilience capacity, resource portfolio, and learning from failure. Some interesting questions include whether and how the psychological capital of emotional network members affects the entrepreneur’s resilience capacity. Are there significant differences in the impact of psychological capital of different social network members on entrepreneurs’ resilience capacity? How does the entrepreneur’s resilience capacity affect their resource portfolio and learning from failure, etc.?

## Author Contributions

LG was responsible for writing the initial draft of the manuscript and putting forward the main propositions. C-FL was responsible for the further modification and improvement of the manuscript. Y-SY was responsible for reviewing and editing the manuscript.

## Conflict of Interest

The authors declare that the research was conducted in the absence of any commercial or financial relationships that could be construed as a potential conflict of interest.
